# Asymptomatic meningitis diagnosed by positron emission tomography in a patient with syndrome of inappropriate antidiuretic hormone secretion: a case report

**DOI:** 10.1186/s13256-021-02956-6

**Published:** 2021-07-22

**Authors:** Masanori Hasebe, Jun Shirakawa, Daisuke Miyashita, Rieko Kunishita, Mayu Kyohara, Tomoko Okuyama, Yu Togashi, Yasuo Terauchi

**Affiliations:** 1 Department of Endocrinology and Metabolism, Graduate School of Medicine, Yokohama City University, Yokohama, Japan; 2 Laboratory of Diabetes and Metabolic Disorders, Institute for Molecular and Cellular Regulation (IMCR), Gunma University, 3-39-15 Showa-machi, Maebashi, 371-8512 Japan

**Keywords:** Hyponatremia, Aseptic meningitis, SIADH, Vasopressin

## Abstract

**Background:**

Syndrome of inappropriate antidiuretic hormone secretion can be caused by arginine-vasopressin-producing tumors or enhanced arginine vasopressin secretion from the posterior pituitary gland due to central nervous system disorders and intrathoracic diseases.

**Case presentation:**

A 53-year-old Asian man was hospitalized with complaints of tremor and hiccups. Laboratory examination revealed findings suggestive of hypotonic hyponatremia due to syndrome of inappropriate antidiuretic hormone secretion. The patient did not complain of headache or photophobia, and showed no signs of meningeal irritation. Positron emission tomography–computed tomography revealed 18F-fluoro-deoxy-glucose accumulation along the cervical spinal cord, based on which the patient was diagnosed as having aseptic meningitis. The hyponatremia was treated successfully by fluid restriction, and optimum plasma sodium concentration was maintained by tolvaptan administration.

**Conclusions:**

This case underscores the need to consider the possibility of mild meningitis as the cause of syndrome of inappropriate antidiuretic hormone secretion in patients without other identifiable cause.

## Background

The concentration of sodium ions in the plasma is maintained within an optimum range concurrently with the extracellular fluid volume, to regulate the plasma osmotic pressure. Hyponatremia can be induced by syndrome of inappropriate antidiuretic hormone secretion (SIADH) and cerebral salt-wasting syndrome (CSWS) [1]. SIADH can be caused by arginine vasopressin (AVP)-producing tumors or enhanced AVP secretion from the posterior pituitary gland. The latter is mostly caused by central nervous system disorders and intrathoracic diseases.

Clinical presentations of SIADH are due to hyponatremia and reduced extracellular fluid osmolality, which results in cerebral edema. The rate and severity of hyponatremia and the degree of cerebral edema determine symptoms. Nonspecific and variable symptoms such as general malaise, loss of appetite, and disturbance of consciousness are manifested by SIADH. Due to the cerebral adaptation, the symptoms sometimes remain mild in the chronic phase despite a serum sodium concentration below 120 mmol/L. The diagnosis of SIADH defined by the Schwartz and Bartter clinical criterion that consists of (1) serum sodium less than 135 mEq/L; (2) decreased plasma osmolality (< 275 mOsm/kg); (3) increased urine osmolality (> 100 moOsm/kg); (4) euvolemia; (5) elevated urine sodium (> 40 mEq/L); (6) absence of adrenal insufficiency, hypothyroidism, cardiac failure, or diuretic use; (7) correction of hyponatremia in response to fluid restriction [2]. Since patients with SIADH commonly show hyponatremia with normal fluid volume, a low serum uric acid level (< 5 mg/dL) and a low plasma renin activity (< 5 ng/mL/hour) can helpful when considering SIADH. AVP can be produced by either a tumor or the neurohypophysis. Because the secretion of AVP from neurohypophysis is heterogeneous, the response of plasma AVP to osmotic stimulation in SIADH was classified into four subtypes [3]. However, the relationship between the clinical symptoms and those different types of osmoregulatory dysfunction remains uncertain.

We report the case of a patient with SIADH associated with meningitis, but in the absence of the typical symptoms of meningitis. The diagnosis of meningitis was made from evidence of cervical myelitis detected on positron emission tomography and computed tomography (PET-CT) images. The hyponatremia was treated successfully by fluid restriction, and optimum plasma sodium concentration was maintained by tolvaptan administration. Thereafter, the dose of tolvaptan could be reduced, and the drug was discontinued altogether after confirming resolution of the abnormalities on cerebrospinal fluid examination. To the best of our knowledge, this is the first reported case of SIADH secondary to meningitis, the latter diagnosed by 18F-fluoro-deoxyglucose (18F-FDG) PET-CT in the absence of the typical clinical symptoms/signs.

## Case presentation

A 53-year-old Asian man presented to us with a 7-day history of fever and tremor, and was admitted to our hospital. The symptoms had persisted/become worse despite treatment with cefditoren pivoxil and acetaminophen for 6 days, and levofloxacin and loxoprofen for 3 days prior to the hospitalization. Two days prior to admission, he had developed generalized malaise, which was sufficiently severe to cause difficulty in walking, and blood tests, chest X-ray examination, whole-body CT, and head CT had been performed at another hospital. The cause of the patient’s symptoms, however, remained unclear. Thereafter, he developed hiccups.

On admission, he complained of persistent generalized fatigue, tremor, and hiccups. He did not have any headache or altered sensorium. His height was 174.5 cm and body weight was 72.0 kg. His body temperature was 36.5 °C at admission, and he remained afebrile while in hospital. His blood pressure was 117/77 mmHg, and pulse rate and respiratory rate were 109 beats/minute and 20 breaths/minute, respectively, both regular rhythm. There were no significant gastrointestinal symptoms, respiratory symptoms, dry skin, or xerostomia. There was no edema or evidence of dehydration. Neurologically, the patient exhibited intention tremor, ataxic gait, and dysmetria on both the finger–nose test and knee–heel tests. Notably, there was no quadriplegia (manual muscle testing revealed full power), sensory disorder, headache, photophobia, or signs of meningeal irritation, including nuchal rigidity, positive Kernig’s sign, or positive Brudzinski’s sign. Laboratory examinations revealed a white blood cell count (WBC) of 8900/μL, red blood cell count of 506 × 10^4^/μL, hemoglobin level of 15.7 g/dL, and platelet count of 20.3 × 10^4^/μL. Serum C-reactive protein level was 0.04 mg/dL. Blood urea nitrogen level was 15 mg/dL, serum creatinine was 0.79 mg/dL, and estimated glomerular filtration rate (eGFR) was 80.3 mL/minute/1.73 m^2^. These results indicated absence of any inflammation or renal dysfunction. Serum electrolyte levels were Na 123 mEq/L, K 3.9 mEq/L, and Cl 86 mEq/L. Plasma osmolarity was 255 mOsm/kg H_2_O, while urinalysis revealed a urinary Na level of 87 mEq/L and urinary osmotic pressure of 691 mOsm/kg H_2_O. The serum ADH level was 1.6 pg/mL, suggesting that inappropriate secretion of ADH was responsible for the excessive excretion of sodium and hypotonic hyponatremia. Measurement of the serum hormone levels showed a plasma adrenocorticotropic hormone (ACTH) concentration of 30.7 pg/mL (normal range 7.4–55.7 pg/mL), cortisol concentration of 14.5 μg/mL (4.5–21.1 μg/mL), thyroid-stimulating hormone (TSH) concentration of 0.397 μIU/mL (0.2–5.0 μIU/mL), free thyroxine (FT4) concentration of 1.38 ng/dL (0.70–1.48 ng/dL), free triiodothyronine (FT3) concentration of 2.29 (1.71–3.71 pg/mL), aldosterone concentration of 98.4 pg/mL (29.9–159 pg/mL), and plasma renin activity of 0.9 ng/mL/hour (0.3–2.9 ng/mL/hour). Thus, there was no evidence of adrenal dysfunction. The physical and laboratory findings were consistent with the diagnosis of SIADH.

**Fig. 1:**
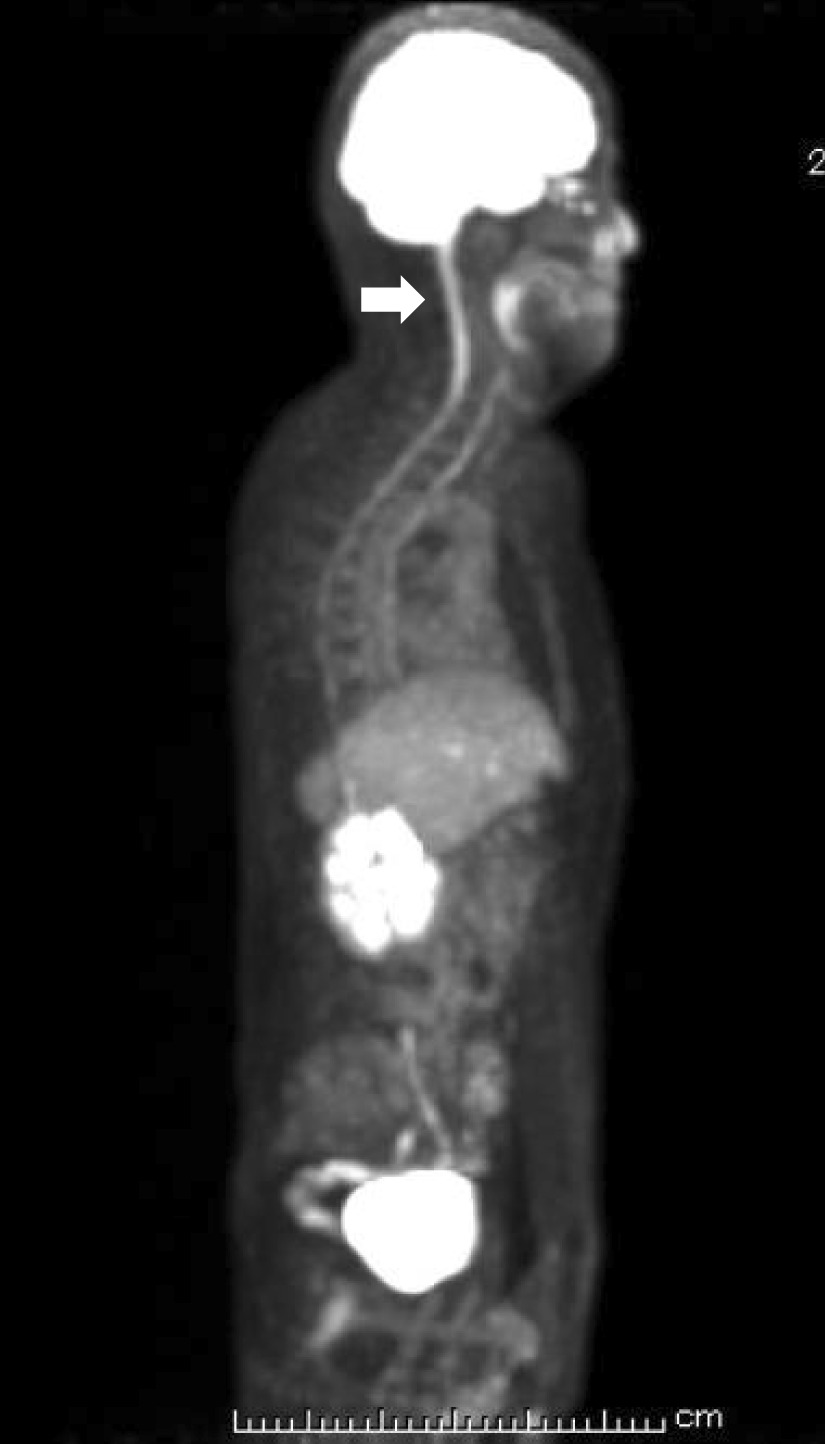
18F-FDG PET-CT imaging. The arrow indicates the abnormal accumulation of FDG along cervical cord

The results of the chest X-ray examination, fundus photographs, contrast-enhanced whole-body CT, contrast-enhanced head CT, and gadolinium-enhanced head magnetic resonance imaging revealed no potential cause for the SIADH. Serum levels of neuron-specific enolase and progastrin-releasing peptide, both small lung carcinoma makers, were within normal limits, and the T-SPOT.TB test, a test for tuberculosis infection, was also negative. 18F-FDG PET-CT revealed an area of tracer accumulation along the cervical cord (Fig. 1, arrow). Based on this PET-CT finding, meningitis was suspected. Examination of the cerebrospinal fluid (CSF) revealed the following findings: cell count 302 cells/3 μL (most were mononuclear cells); total protein 211 mg/dL; albumin 1323 µg/mL; glucose 49 mg/dL; β2-microgroblin 8447 ng/mL (440–1240 ng/mL); immunoglobulin G (IgG) 16.6 mg/dL (1–3 mg/dL); IgG index 0.61 (< 0.73); adenosine deaminase 10.2 U/L (< 9.0 U/L); myelin basic protein 175.1 pg/mL (< 102 pg/mL); no oligoclonal band detected. No organisms were detected on Gram staining or culture of the CSF. We considered these findings as being diagnostic of aseptic meningitis or partially treated meningitis, in particular, the findings that the cells were predominantly composed of mononuclear cells and that there was no apparent evidence of bacterial infection or underlying autoimmune diseases such as systemic lupus erythematosus and Behçet’s disease as the cause of the meningitis/encephalitis.

**Fig. 2:**
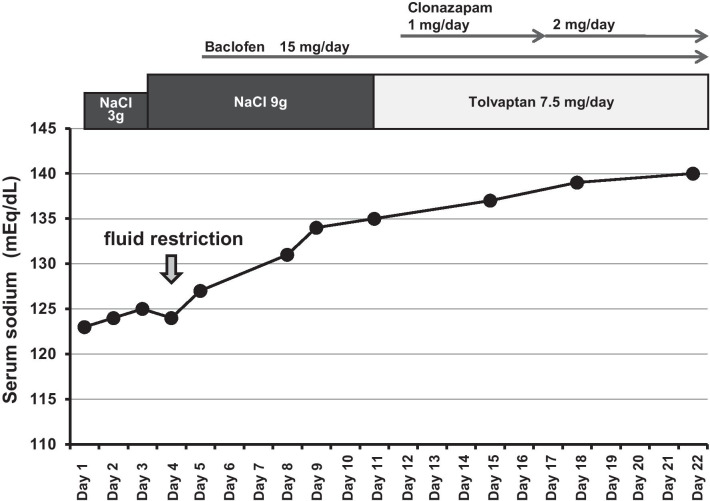
Clinical course. The arrow indicates fluid restriction to 800 mL/day. The serum sodium level improved after the fluid restriction and remained stable after the start of tolvaptan administration. Baclofen 15 mg/day and clonazepam 1 mg/day were used as symptomatic treatments for the hiccups and tremor; the dose of clonazepam was subsequently increased to 2 mg/day

Although both intravenous infusion of physiological saline for a day and oral administration of sodium chloride were started, the serum sodium level remained below 125 mEq/L, and improved only after fluid restriction to 800 mL/day (Fig. 2, arrow) and initiation of treatment with tolvaptan 7.5 mg/day (Fig. 2). Thereafter, the serum sodium level remained stable (at least until 4 months after admission). Aseptic meningitis was suspected as the cause of the tremor and hiccups. Baclofen 15 mg/day and clonazepam 1 mg/day were administered as symptomatic treatment for the hiccups and tremor, respectively. The hiccups rapidly resolved in response to this treatment, and the tremor tended to improve after the dosage of clonazepam was increased to 2 mg/day. A repeat CSF examination performed on day 22 before discharge of the patient confirmed a reduction of the CSF cell count to 125/3 μL from 302/3 μL, the latter count recorded in the earlier examination. Thus, our patient showed spontaneous partial remission of the aseptic meningitis. The administration of tolvaptan, baclofen, and clonazepam was gradually reduced after discharge and discontinued by a month after discharge. At 4 months after discharge, the symptomatic improvement was maintained. Throughout the observation period of 4 months, normonatremia was maintained without further treatment with tolvaptan.

## Discussion

SIADH is diagnosed when blood and urine examinations reveal evidence of euvolemic hypotonic hyponatremia and an inappropriately high urine osmolality due to excessive sodium excretion, in the absence of evidence of renal or adrenal dysfunction [4, 5]. Excessive release of AVP relative to the osmotic pressure from the pituitary by excessive stimulation of the pressure receptors in the carotid sinus or from AVP-producing tumors, including lung cancer, causes SIADH. In the case of ectopic expression of AVP, AVP-producing tumor cells are generally detected in the lung, pancreas, or other tissues. In addition to intrathoracic diseases such as pneumonia and pulmonary tuberculosis, SIADH could often be induced by central nervous system (CNS) causing meningeal stimulation, including subarachnoid hemorrhage, meningitis, and cerebral infarction. The mechanism of AVP hypersecretion from the posterior pituitary secondary to CNS disorder is, however, not clear. In the patient reported herein, the SIADH was considered to have developed as a complication of aseptic meningitis or partially treated meningitis, the diagnosis of which could only be made by PET-CT, as there were no clinical signs/symptoms of meningitis.

In a previous study conducted in infant patients, urinary vasopressin levels were significantly higher in patients with bacterial meningitis than in those with aseptic meningitis [6]. In the patient reported herein with aseptic meningitis, the serum AVP level was relatively low (1.6 pg/mL). Bacterial meningitis generally provokes more severe inflammation and meningeal irritation as compared with meningitis caused by viruses, although definitive differentiation between the two totally relies on examination of the CSF [7]. It is possible that the severity of meningitis is correlated with the AVP secretion levels in SIADH. Patients with meningitis often exhibit hyponatremia with increased urinary sodium excretion because of SIADH and CSWS. No clinical symptoms of CSWS, including dehydration or body fluid loss due to excessive urinary volume and urinary sodium excretion, were observed in our case. In the case of meningitis caused by *Pseudomonas aeruginosa*, it is known that the patients develop SIADH coinciding with the active phase of meningitis, and that the serum sodium levels become normal with the resolution of meningitis [8]. Other reports of cases of aseptic meningitis with mild encephalitis/encephalopathy associated with a reversible splenial lesion (MERS) also showed resolution of the hyponatremia with improvement of the disease. Our patient also showed restoration of the serum sodium levels to normal range after remission of the aseptic meningitis, indicating that the meningitis was indeed the cause of SIADH.

None of the clinical history, physical examination findings, or findings of biochemical/radiological analysis suggested the involvement of drugs, malignant tumors, intrathoracic diseases, or intracranial lesions in the development of SIADH in our patient. On admission, he had no headache, fever, or any signs of meningeal irritation. 18F-FDG PET-CT identified a spinal lesion, with increased accumulation of FDG in the upper spinal canal. CSF examination revealed findings consistent with the diagnosis of aseptic meningitis or partially treated meningitis. Abnormal FDG accumulation on PET-CT has been reported in patients with malignant tumors or tuberculous meningitis who present with the typical symptoms of meningitis such as fever, headache, nausea, or meningeal irritation [9–12]. We could not identify any case report in the literature in which the diagnosis needed PET-CT, as in our patient. Patients with acquired immune deficiency syndrome presenting with *Cryptococcus neoformans* meningitis show no typical symptoms of meningitis such as headache, disturbance of consciousness, or clinical features of meningeal irritation [13]. However, in these patients, cryptococcal antigen is identified in the CSF, although CSF examination may reveal no biochemical abnormalities. It has been thought that the signs of meningeal irritation are derived from increased intracranial pressure [14]. In our patient, the typical symptoms and signs of meningeal irritation may not have manifested, because the cell count in the CSF and the CSF pressure showed only mild-to-moderate elevation. Hitherto, most cases of SIADH secondary to meningitis have been reported in cases with the typical symptoms and signs of meningitis [15]. Idiopathic forms of SIADH after a series of examinations have often been reported in patients, especially older patients, with hyponatremia [16, 17]. It is conceivable that, in a considerable number of cases with presumed idiopathic SIADH, the diagnosis of asymptomatic meningitis or partially treated meningitis may have been missed.

In one report of a case of SIADH secondary to bacterial meningoencephalitis associated with traumatic brain injury, treatment with tolvaptan promptly improved the hyponatremia, despite the poor initial response to electrolyte infusion [18]. Aseptic meningitis often shows spontaneous recovery and carries a better prognosis as compared with bacterial meningitis. Therefore, we treated the SIADH in our patient with tolvaptan, which resulted in resolution of the hyponatremia. Because tolvaptan is not yet approved for the treatment of SIADH in Japan at present, appropriate informed consent was obtained from our patient for the treatment. Hiccups has been reported in a case of aseptic meningitis associated with systemic lupus erythematosus [19]. In that case, the symptom resolved immediately after the start of treatment of the meningitis with prednisolone. We thought that the hiccups in our patient could also have been caused by the aseptic meningitis.

## Conclusions

In the present case, the diagnosis of meningitis was made by PET-CT, and we succeeded in the conservative treatment of SIADH with tolvaptan. Empiric antimicrobial therapy is required immediately in cases with suspected bacterial meningitis. Our case report highlights the importance of considering mild-to-moderate meningitis in the differential diagnosis in patients with SIADH without any apparent underlying cause. Our report also underscores the usefulness of 18F-FDG PET-CT imaging for the detection of meningitis in a patient without signs of meningeal irritation. It should be noted that PET-CT is not generally recommended for the diagnosis of meningitis from the viewpoint of the cost-effectiveness of screening. Therefore, we should review the physical, neurological, and biochemical findings carefully and consider the results of CSF examination in SIADH patients with suspected mild meningitis.

## Data Availability

Not applicable.

## Abbreviations

SIADH: Syndrome of inappropriate antidiuretic hormone secretion
AVP: Arginine vasopressin
CSWS: Cerebral salt-wasting syndrome
PET-CT: Positron emission tomography and computed tomography
FDG: 18F-fluoro-deoxy-glucose
CSF: Cerebrospinal fluid
